# First person – Sutirtha Lahiri

**DOI:** 10.1242/bio.058851

**Published:** 2021-06-22

## Abstract

First Person is a series of interviews with the first authors of a selection of papers published in Biology Open, helping early-career researchers promote themselves alongside their papers. Sutirtha Lahiri is first author on ‘Convergent acoustic community structure in South Asian dry and wet grassland birds’, published in BiO. Sutirtha is a research assistant in the lab of Dr Anand Krishnan at the Indian Institute of Science Education and Research (IISER) Pune, India, investigating whether biogeographically distinct grasslands display convergent acoustic community structure.


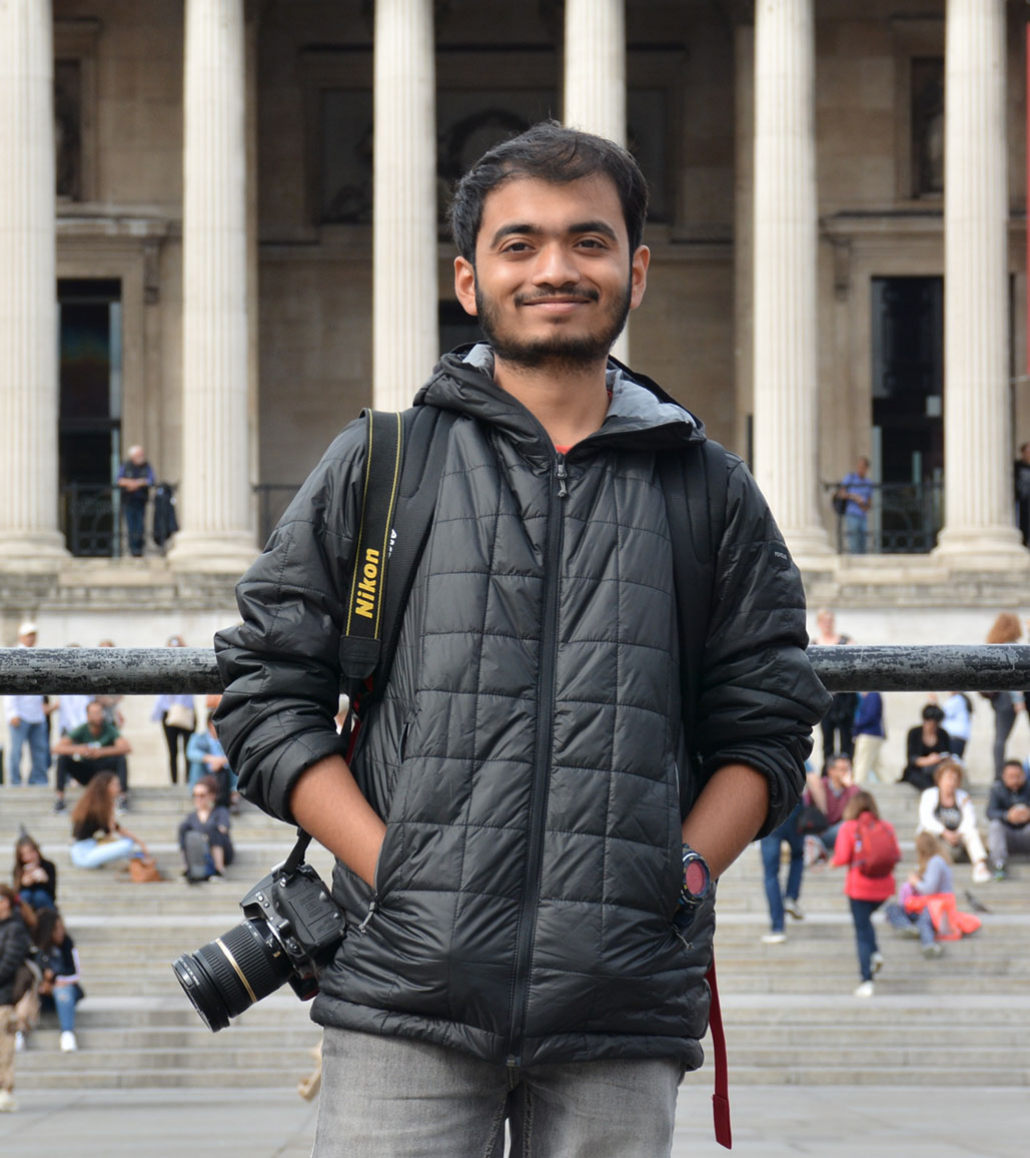


**Sutirtha Lahiri**

**What is your scientific background and the general focus of your lab?**

I completed a master's in wildlife science from the Wildlife Institute of India, Dehradun, India. Here, I studied how sympatric drongos (a group of passerines) partition their acoustic niche in a tropical rainforest in Assam, India. Following my interest in bioacoustics, I joined Dr Anand Krishnan's lab at IISER Pune. The lab studies bioacoustics, using natural sounds to understand behaviour, ecology and evolutionary processes in diverse taxa. We integrate data collected from field, lab and museums at multiple scales, ranging from individual species to entire communities.

**How would you explain the main findings of your paper to non-scientific family and friends?**

Our study was conducted in two distinct grassland habitats – a dry grassland in western India and a wet grassland in eastern India. We used acoustic recorders to study singing birds (the acoustic community) in these two grassland types, and find that birds tend to separate – or have a distinct position – in the acoustic space (overdispersion), which may help avoid masking interference from each other's signals. Interestingly, in spite of being distinct regions, the two grasslands show a convergent pattern, suggesting that each species in one habitat has a counterpart in the acoustic space of the other. The wet grasslands in east India are occupied by an expansion of birds belonging to the Sylvioidea clade (which represents babblers, warblers, bulbuls and larks). We suggest that signals of this clade have converged with those of species in dry grasslands. Thus, the similar pattern between the two habitats may result from species’ songs converging to occupy the acoustic space.

**What are the potential implications of these results for your field of research?**

Not only does our study shed light on interesting convergent patterns of acoustic communities in two distinct grassland habitats, our study is also one of the first of its kind to use bioacoustic monitoring in these ecosystems. Passive acoustic monitoring allows us to collect data in otherwise difficult to access habitats. Many grassland species are of high conservation concern owing to their specialized habitat requirements and the threats facing these habitats. Studying acoustic communities thus not only provides input into how these grassland communities assemble, but also will enable us to track them year after year to quantify how the community changes. This will provide valuable conservation information all the way from individual threatened species to the entire community, as an early warning system of ecological change.

**Figure:**
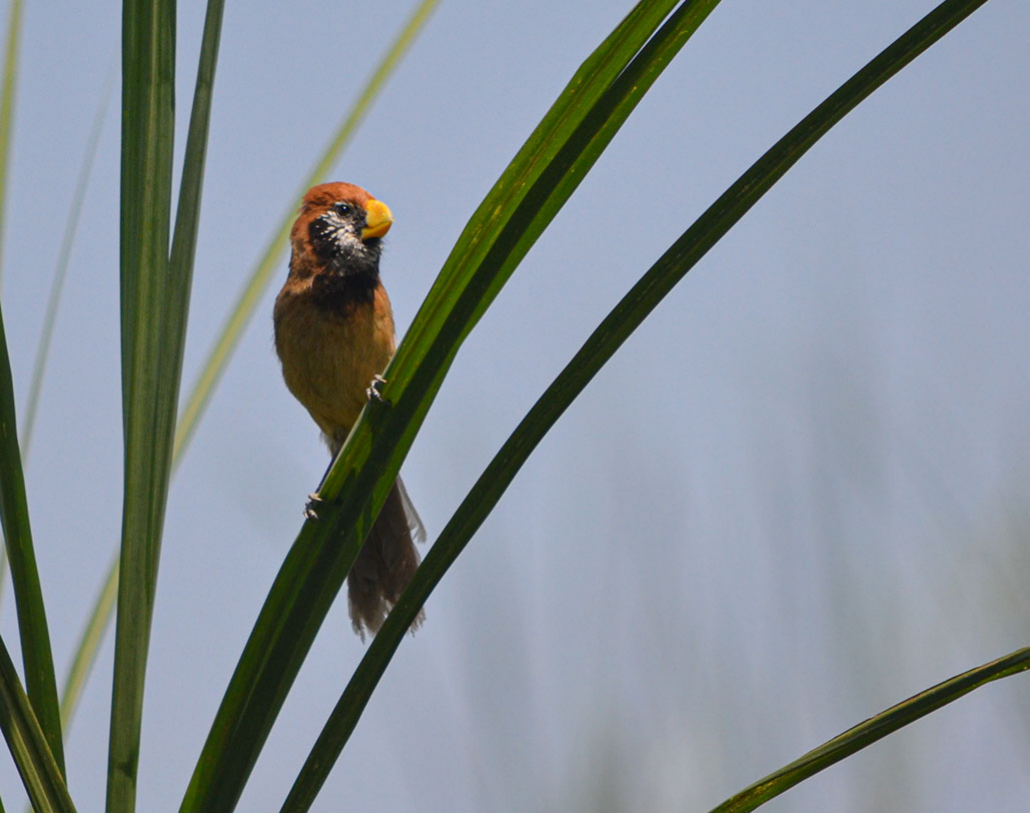
**The black-breasted parrotbill (*Paradoxornis flavirostris*) is an endemic bird of the wet grasslands.** A species of conservation concern, the parrotbill went undetected for decades in the 1900s before being documented in some small and disjointed pockets of northeast India. Photo credit: Taksh Sangwan.

“…our study is also one of the first of its kind to use bioacoustic monitoring in these ecosystems.”

**What has surprised you the most while conducting your research?**

Understanding how a particular clade of birds – the Sylvioidea – expanded to occupy acoustic space in the grassland in eastern India was definitely exciting as it corroborates with the high diversity of this clade in that part of the country. Additionally, being able to record and document bird species that are otherwise very difficult to see in the dense grassland habitats were also a highlight of the research. For many of these species, our data is some of the first quantitative information regarding their occurrence, as they have evaded ornithologists owing to their limited population and the availability of such habitats. It was exciting that our idea to study some of these species using acoustics paid off! D'Ering Wildlife Sanctuary, a tall grassland habitat, is imperilled by inundation by the river due to mega dam construction upstream as well as the effects of climate change that are changing rainfall patterns and the river flow dynamics, which are likely to have an impact on the longevity of these unique grassland habitats.

“For many of these species, our data is some of the first quantitative information regarding their occurrence, as they have evaded ornithologists owing to their limited population and the availability of such habitats.”

**What, in your opinion, are some of the greatest achievements in your field and how has this influenced your research?**

One of the most influential works on acoustic communities come from the foundational studies by Dr David Luther (Luther, 2009), which have influenced much of our work. Additionally, the development of low-cost, lightweight passive recorders that have the potential to record in adverse weather conditions for long hours has revolutionised the field of bioacoustics, and it promises for long-term monitoring of species and habitats. We are hopeful that with the accessibility to even cheaper recorders, we can expand our study to larger spatial and temporal scales, and thus ask more interesting questions along the way!

**What changes do you think could improve the professional lives of early-career scientists?**

Accessibility to resources and events that help to reduce barriers to science is one of the biggest factors that can help early-career scientists. By accessibility, I mean access to scientific papers, conferences, workshops. This is of special importance to scientists from the global south, where money poses a great challenge to access these resources. In that regard, Open Access journals (such as Biology Open) and the ever-increasing utility of digital platforms for conducting conferences and workshops, can go a long way in ensuring this.

**What's next for you?**

I will be joining the University of Minnesota, USA in the fall of 2021 for a PhD.
